# Myocardial Infarction With Nonobstructive Coronary Arteries With Coronary Microvascular Dysfunction Associated With Systemic Sclerosis: Case Report

**DOI:** 10.1155/crvm/9472631

**Published:** 2026-05-22

**Authors:** Yuya Ohga, Tatsuya Nishikawa, Akeo Hirai, Go Nakano, Ryota Yoshioka, Atsushi Iseki, Tatsuya Matsubara, Hibiki Kadohara, Motohiro Shingu, Yuto Osumi, Kenta Ishibashi, Toshimitsu Ishii, Mana Hiraishi, Mitsuo Kinugasa, Yasutaka Hirayama, Kouichi Tamita

**Affiliations:** ^1^ Department of Cardiovascular Medicine, Akashi Medical Center, Akashi, Japan, amc1.jp; ^2^ Department of Clinical Engineering, Akashi Medical Center, Akashi, Japan, amc1.jp

**Keywords:** CMD, MINOCA, systemic sclerosis

## Abstract

**Background:**

Myocardial infarction with nonobstructive coronary arteries (MINOCA) accounts for a meaningful proportion of acute myocardial infarction presentations and encompasses heterogeneous mechanisms. Coronary microvascular dysfunction (CMD) is a major cause of MINOCA and can be invasively characterized using coronary physiology indices, including the index of microvascular resistance (IMR) and coronary flow reserve (CFR). Systemic sclerosis (SSc) is associated with small‐vessel vasculopathy and may predispose individuals to CMD. However, MINOCA attributable to SSc‐related CMD confirmed by invasive coronary function testing and cardiac magnetic resonance (CMR) is not widely reported.

**Case Presentation:**

A 76‐year‐old woman with SSc and interstitial pneumonia presented with persistent chest pain. Electrocardiography revealed new T‐wave inversions in leads V1–V4, and echocardiography demonstrated mid‐anterior left ventricular asynergy. Troponin T was elevated (0.206 ng/mL). Emergency coronary angiography showed no obstructive epicardial stenosis, but left ventriculography confirmed regional wall‐motion abnormality. CMR demonstrated base‐to‐mid anteroseptal dysfunction with increased native T1, T2, and extracellular volume fraction, supporting MINOCA. Transient systemic inflammation was observed (peak C‐reactive protein 14.69 mg/dL; white blood cell count 24,600/*μ*L) without evidence of infection and it resolved spontaneously. Six weeks later, acetylcholine provocation testing was negative for epicardial spasm, but invasive physiological assessment identified CMD (IMR 30.5; CFR 1.7). The patient was treated with a calcium‐channel blocker and nicorandil. At 3‐month follow‐up, symptoms, electrocardiographic changes, and CMR abnormalities normalized, which indicated a reverse of the ischemic injury. No recurrence occurred during 1 year of follow‐up.

**Conclusion:**

SSc‐associated MINOCA, possibly driven by CMD, was diagnosed by comprehensive invasive coronary function testing and CMR. Serial CMR may help to document reversibility of microvascular ischemic injury and guide vasodilator therapy and follow‐up.

## 1. Background

Myocardial infarction with nonobstructive coronary arteries (MINOCA) has long been of great interest to cardiologists. MINOCA is a clinical situation in which a patient presents with symptoms suggestive of acute coronary syndrome. They demonstrate troponin elevation, and there are nonobstructive coronary arteries at the time of coronary angiography (defined as coronary artery stenosis < 50% in any major epicardial vessel) [1]. The prevalence of MINOCA among patients with acute myocardial infarction has been reported as 6% [2], although it seems to vary across studies.

Coronary microvascular dysfunction (CMD) is a major cause of MINOCA. It can now be diagnosed by functional coronary angiography using pressure wire. CMD is assessed by two markers; one is the index of microvascular resistance (IMR), which indicates the impedance of microcirculation, and the other is coronary flow reserve (CFR), which indicates the capability of microvascular expansion. The clinical presentation and the causes of CMD have been related to a wide spectrum of backgrounds. Here, we report the case of a patient with MINOCA that was potentially associated with CMD related to systemic sclerosis (SSc). The MINOCA and CMD were diagnosed by functional coronary angiography and cardiac magnetic resonance (CMR).

## 2. Case

**Figure 1:**
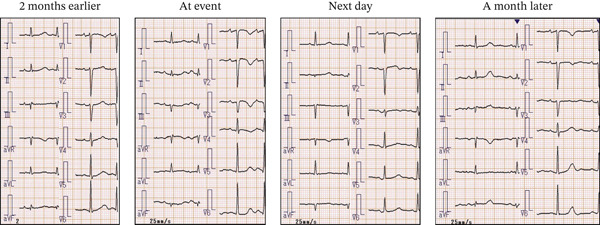
Electrocardiogram with time course.

**Figure 2:**
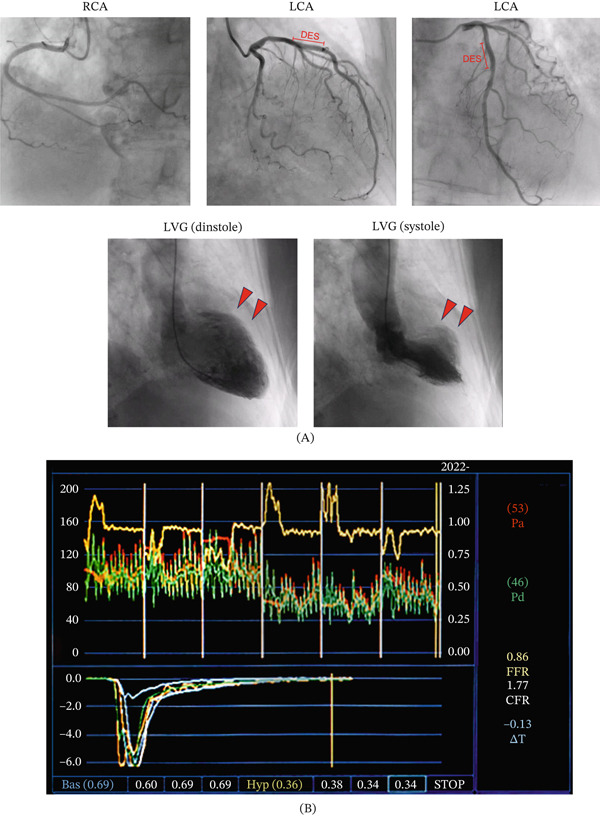
Images of cardiac catheterization. (A) Emergent coronary angiography and left ventriculogram at the event of MINOCA. The red arrowhead shows regional wall‐motion abnormality (Segments 1 and 2). DES, drug eluting stent. (B) Measurement of coronary microvascular examination by pressure wire. CFR and the index of microcirculatory resistance (IMR) were measured in the left anterior descending artery using the RadiAnalyzer Xpress and PressureWire X (Abbott). The bolus thermodilution method was used, and hyperemia was induced by continuous intravenous infusion of adenosine.

A 76‐year‐old woman was brought to our hospital by ambulance for chest pain. The chest pain started at approximately 21:00 but was still present when she woke up the next morning. Her vital signs were as follows: heart rate 96 bpm, blood pressure 155/67 mmHg, SpO_2_ 100% (room air), and respiration rate 24/min. Electrocardiography showed sinus rhythm with negative T wave in V1–4, which had not been observed in the most recent previous data (2 years previously) (Figure 1). Echocardiography showed regional wall‐motion abnormality at the mid‐anterior wall of the left ventricle. There was apparently no obvious valve disease. Laboratory data showed elevation of troponin T (0.206 ng/mL). Under the diagnosis of non‐ST elevated myocardial infarction, the patient underwent emergency coronary angiography, which showed no obstructive epicardial stenosis. However, left ventricular angiography showed regional wall‐motion abnormality on Segments 1 and 2 (Figure 2A). The patient had undergone percutaneous coronary intervention for chronic coronary syndrome 5 years earlier, during which a notable stenosis in the proximal left anterior descending artery had been treated with a drug‐eluting stent. At the time of the present MINOCA event, however, coronary angiography showed no evidence of in‐stent restenosis, stent thrombosis, edge‐related disease, or other subtle angiographic abnormalities. Unfortunately, intravascular imaging, such as optical coherence tomography or intravascular ultrasound, was not performed in this case because there were no signs of stenosis by angiography.

**Figure 3:**
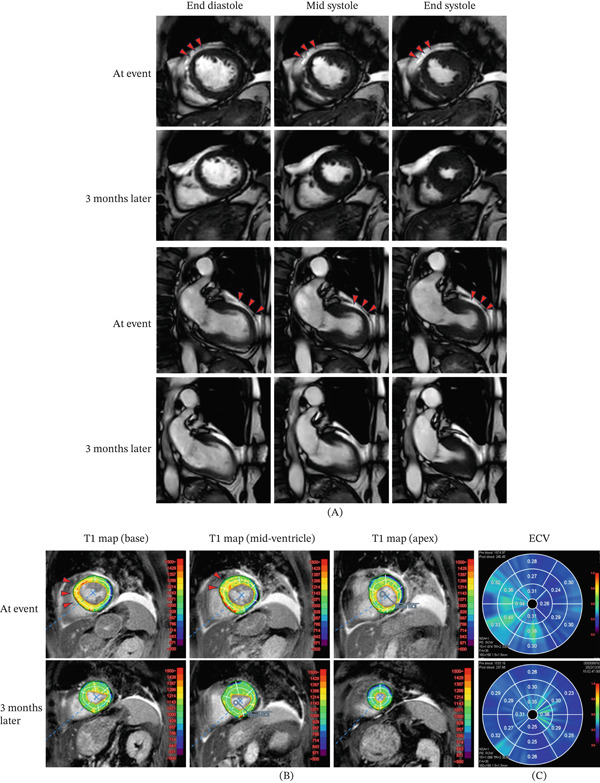
Cardiac magnetic resonance images with time course. (A) Cine MRI with time course. The red arrowheads indicate the area of severe hypokinesis in the left ventricle. (B) T1 mapping at the event and 3 months later. The red arrowheads show the area with high native T1. (C) Bull′s eye images of extracellular volume fraction at the event and then at 3 months later.

In addition, CMR also showed regional wall‐motion abnormality from the basal to mid anteroseptal segments, which was accompanied by elevated native T1 values (basal: 1221 ms; mid‐ventricular: 1152 ms; local normal range: 973 ± 47 ms) and increased extracellular volume fraction (basal: 32%; mid‐ventricular: 36%) (Figure 3). No late gadolinium enhancement (LGE) was observed in the corresponding region. The patient was therefore diagnosed as having MINOCA. On the next day, the ST change of electrocardiography was improved to baseline. However, her C‐reactive protein (CRP) and white blood cell (WBC) count elevated to the peak level: 14.69 mg/dL (normal range, < 0.14 mg/dL) and 24,600/*μ*L (normal range, 3300–8600/*μ*L), respectively. On the fourth day, CRP and WBC were decreased to 7.49 mg/dL and 10,880/*μ*L, respectively, without any treatment such as antibiotics or additional immunosuppression therapy. At that time, the exact reason for this transient inflammation was unclear. It was not considered to be due to infection, but rather due to a potential autoimmune modulation.

Regarding past history, the patient had been diagnosed with SSc with interstitial pneumonia about 7 years previously. Steroid and immune suppression therapy (tacrolimus) was started 2 years after the diagnosis. Two months after the steroid introduction, she was seen to have coronary artery disease. The patient had no habit of smoking or consuming alcohol.

Six weeks after the MINOCA event, we re‐evaluated its underlying mechanism by performing invasive coronary angiography with physiological assessment. Physiological testing was conducted in accordance with the recommendations of European and Japanese guidelines [3, 4]. CFR and the index of microcirculatory resistance were measured using the bolus thermodilution method with the RadiAnalyzer Xpress and PressureWire X system (Abbott Vascular, St. Paul, Minnesota, United States). Epicardial spasm during acetylcholine provocation testing was defined as > 90% transient coronary vasoconstriction accompanied by signs or symptoms of myocardial ischemia, such as chest pain and ST‐segment changes. Acetylcholine provocation testing was performed in both the right coronary artery (25 and 50 *μ*g bolus injections) and the left coronary artery (25, 50, and 75 *μ*g bolus injections) without prior nitrate administration. Hyperemia was induced by continuous intravenous infusion of adenosine at 0.18 mg/kg/min. The results of acetylcholine provocation test showed no obvious evidence of vasospasm in the epicardial coronary artery. However, physiological examination with the left anterior descending artery as a target vessel showed CMD with high IMR 30.5 (normal range, < 25) and low CFR 1.7 (normal range, > 2.5) assessed by Radi Analyzer Xpress instrument with PressureWire X (Abbott Vascular) (Figure 2B).

We therefore determined the cause of MINOCA to be probably CMD related to SSc, but maintained that coexistence of microvascular spasm could not be completely denied. As a treatment, we administered a Ca blocker and nicorandil, predominantly to target microvascular vasoconstriction [3].

After 3 months, CMR was followed up. The regional wall‐motion abnormality, abnormality of native T1 (1010 ms), T2‐STIR, and extracellular volume fraction (29%) were all normalized (Figure 3), indicating that a regional and transient ischemia was caused by CMD. There was no LGE in the area. The ST‐T changes in the 12‐lead electrocardiogram were also normalized at 3 months after the event. Medical treatment comprising Ca blocker and nicorandil was continued. One year after the event, there have been no recurrences of MINOCA events or chest pain.

## 3. Discussion

We reported a case of MINOCA with regional myocardial damage on chronic global CMD caused by SSc. Notably, the patient′s CMD was invasively diagnosed by IMR and CFR, and MINOCA was assessed by CMR.

SSc is a severe systemic autoimmune disease that is characterized by small vascular impairment with endothelial damage resulting in vasculopathy and fibrosis including the heart. CMD in patients with SSc has been widely reported. Most of these were noninvasive CFR assessment by echocardiography or histopathological assessment [5, 6]. Definite diagnosis of MINOCA and CMD by functional coronary angiography has not been reported in the literature. Regarding SSc, abnormal vasoreactivity reportedly induces transient coronary artery spasm and repeated focal ischemia [7]. Finally, accumulation of the mechanism leads to concentric intimal hypertrophy of small coronary arteries, and this is followed by cardiomyocyte damage and myocardial fibrosis [7]. The relationship between CMD and SSc has been known for decades: coronary reserve impairment in patients with SSc was revealed by assessment of coronary sinus blood flow back in 1985 [8]. Elsewhere, the autopsied myocardia of patients with SSc showed a higher prevalence of patchy fibrosis than a control group, and it was assessed as the result of myocardial microvascular vasospasm, also called “myocardial Raynaud′s phenomenon” [9].

In this case, we could not determine a specific mechanism behind the acute cardiac ischemic injury. However, a possible mechanism could be regional microvascular spasm or acute microvascular obstruction relating to inflammation caused by SSc. At the point of the MINOCA event, SSc might have been acutely worsening: The patient′s CRP and WBC were extremely and transiently elevated, which might have been the result of SSc worsening. CRP has been reported to be elevated in patients with SSc with interstitial lung disease, but usually CRP is not as highly elevated as in our patient [10]. In the present case, there were no other progressive symptoms of the skin, the lungs, or of heart failure. Unfortunately, we did not examine biomarkers such as specific antibodies in this case, so we cannot prove the supposed SSc activity. However, the electrocardiogram change was improved the next day, and regional wall‐motion abnormality soon improved, so we suggest that there may have been an additional factor as well as the chronic state of CMD. Considering the cumulative evidence, we suggest that our patient′s microvascular coronary vasospasm may have been related to the MINOCA. The acetylcholine provocation test was negative, but if only a small regional microvascular vasospasm was induced, it might not be detectable by angiography or electrocardiogram, and it might be asymptomatic.

Reported causes of MINOCA include plaque rupture, plaque erosion, in situ thrombosis, spontaneous coronary artery dissection, supply‐demand mismatch, epicardial coronary vasospasm, and CMD [11]. In an earlier report, coronary vasospasm was said to be a common cause of MINOCA. Coronary vasospasm was diagnosed in 46.2% of patients with MINOCA undergoing provocative testing. Among those with a positive test, epicardial spasm was detected in 64.9% and microvascular spasm in 35.1% [12]. Patients with various backgrounds may have microvascular spasms, such as those with SSc. Recently, there has been progress in methodology of measuring physiological markers and understanding the pathology of CMD, so further research into the prevalence, clinical presentation, treatment and prognosis of the SSc with CMD could inform the treatment strategy for affected patients.

A limitation of this case is that intravascular imaging was not performed during the initial coronary angiography. The 2023 ESC Guidelines for the management of acute coronary syndromes state that intravascular imaging may be useful when there is uncertainty regarding the culprit lesion (Class IIb) [1]. The initial angiographic findings were not considered ambiguous at the time, but intravascular imaging might have provided additional diagnostic information in the present case. Furthermore, several potential differential diagnoses remained, including coronary thrombosis, Type 2 myocardial infarction, regional myocarditis, and takotsubo syndrome. However, an epicardial coronary culprit lesion appears less likely because the coronary perfusion territory did not correspond to the regional myocardial abnormalities observed on CMR. In contrast, myocarditis and takotsubo syndrome could not be completely excluded, as no single cardiovascular imaging modality can definitively rule them out in all cases. Nevertheless, the absence of LGE on CMR is somewhat atypical for myocarditis, and the electrocardiographic time‐course was not typical of takotsubo syndrome. Patients with severe organ involvement in SSc such as the heart, the lungs, and gastrointestinal organs reportedly have poorer prognoses than those without [13]. Cases like ours therefore require careful follow‐up by a multidisciplinary medical team.

## Funding

No funding was received for this manuscript.

## Consent

The authors confirm that patient consent is not applicable to this article. This is a retrospective case report using deidentified data, so the IRB did not require consent from the patient.

## Conflicts of Interest

The authors declare no conflicts of interest.

## Data Availability

Data sharing is not applicable to this article as no datasets were generated or analyzed during the current study.
